# The tympanic covering layer contributes to basilar membrane elasticity potentially influencing human frequency resolution and speech perception

**DOI:** 10.1111/joa.70114

**Published:** 2026-02-11

**Authors:** Wei Liu, Claudia Steinacher, Alexander Glueckert, Rudolf Glueckert, Hao Li, Karin Staxäng, Anneliese Schrott‐Fischer, Hanif M. Ladak, Sumit Agrawal, Helge Rask‐Andersen

**Affiliations:** ^1^ Department of Surgical Sciences, Otolaryngology and Head and Neck Surgery Uppsala University Uppsala Sweden; ^2^ Inner Ear Laboratory, Department of Otorhinolaryngology Medical University Innsbruck Innsbruck Austria; ^3^ The Rudbeck TEM Laboratory, BioVis Platform Uppsala University Uppsala Sweden; ^4^ Department of Medical Biophysics Western University London Ontario Canada; ^5^ Department of Electrical and Computer Engineering Western University London Ontario Canada; ^6^ Department of Otolaryngology‐Head and Neck Surgery Western University London Ontario Canada

**Keywords:** basilar membrane, cochlea, elastin, EMILIN‐2, human, tympanic covering layer

## Abstract

EMILIN family extracellular glycoproteins in the basilar membrane (BM) have so far only been identified in the mouse. It may influence the stiffness and extracellular filamentous architecture essential for cochlear frequency tuning (Russell et al., 2020, *Science Advances*, 6, eaba2634). In our study, we explored whether elastic components, such as EMILIN‐2 (elastin microfiber interface‐located protein 2) and elastin, are also present in the human BM. In addition, we analyzed the fine morphology of the BM and tympanic covering layer (TCL) at different frequency locations using light, transmission electron, and scanning electron microscopy (LM, TEM, and SEM) in well‐preserved human cochlear specimens. Tonotopic estimations were made from model extrapolations using synchrotron radiation phase‐contrast imaging (SR‐PCI) and 3D reconstruction of matched cochleae with delineated octave bands constructed using Greenwood's formula (Greenwood, 1961, *Journal of the Acoustical Society of America*, 33, 1344). Our immunohistochemistry and gene RNA sequencing (RNA‐seq) data support that EMILIN‐2 and elastin are expressed in the human BM. Expression pattern of both proteins that influence BM architecture vary along the frequency range. TEM analysis suggests that TCL cells are actively involved in the deposition of the amorphous substance and fibrillary network, conceivably serving to assemble extracellular and elastic constituents along the BM. Thus, our findings suggest that TCL cells may play an important functional role in human cochlear tuning, particularly at low frequencies potentially linked to our remarkable speech and music perception.

## Graphical abstract

In this study we show for the first time that the human basilar membrane contains elastin produced by the so‐called tympanic covering layer. It is believed to play an important functional role in human cochlear tuning, particularly low frequencies linked to our remarkable speech and music perception.

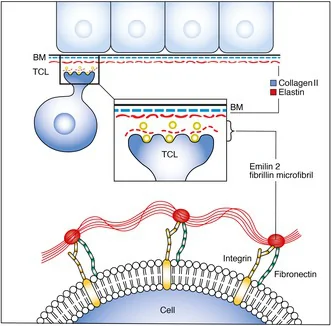

## 1 INTRODUCTION

The basilar membrane (BM) plays a pivotal role in the frequency selectivity of the cochlea (Dallos et al., 1974; Helmholtz, 1863; Johnstone & Boyle, 1967; Tonndorf & Khanna, 1968; Zwislocki, 1948). Different sound stimuli produce characteristic frequency‐specific traveling waves along the BM that reach maxima at different locations (Békésy, 1960). Frequency resolution and sensitivity depend on the physical properties of the BM, among which elasticity, which was found to increase 100 times from the base to apex in human cadaver ears (Békésy, 1960).

Recent studies suggest that the human apical cochlea possesses particular mechanical and biophysical features that could partly explain its optimization for decoding speech under various conditions (Giese et al., 2024; Li et al., 2025). Previously, we gathered information on the human BM using light and electron microscopy in combination with immunohistochemistry (Liu, Atturo, et al., 2015). In this study, we extended our research to include elastin microfiber interface‐located protein 2 (EMILIN‐2) and elastin. EMILIN family extracellular glycoproteins have been shown to be present in the mouse BM, where they may influence its filamentous architecture and stiffness, which are important for cochlear frequency tuning (Russell et al., 2020).

We analyzed the human BM microanatomy and expression of EMILIN‐2 at different frequency locations (estimated from model extrapolations using synchrotron phase‐contrast imaging and 3D reconstruction). The results confirmed that there are fundamental differences in the morphology of the human BM in high‐ and low‐frequency regions. Basally, the BM consists of a pars arcuata and pars pectinata, similar to that of earlier investigated animals, such as the mouse and some Talpa species (Russell et al., 2020). Part tecta corresponds to pars arcuata.

Our findings suggest that TCL cells may play a fundamental role in the production of elastic components in the human BM. EMILIN‐2 was expressed in the tympanic covering layer (TCL) and in an amorphous ground substance (GS), also expressing elastin. Unlike data from earlier human studies, these results suggest that structural components providing elasticity to the BM may play a role in human cochlear frequency tuning and speech perception.

## 2 MATERIALS AND METHODS

### 2.1 Antibodies and immunohistochemistry

The EMILIN‐2 antibody used was a polyclonal rabbit Ab (HPA040739; Sigma). The elastin antibody was a mouse monoclonal Ab (MAB2503; Millipore, Billerica, MA, USA). The antibody against laminin‐β2 was a monoclonal antibody from rat (Cat. No. 05‐206, dilution 1:100; Millipore). It recognizes and is specific to the β2 chain laminin. The type IV collagen antibody was a goat polyclonal antibody (Cat. No. AB769, dilution 1:10; Millipore). It has less than 10% cross‐reactivity with collagen types I, II, III, V, and VI. The type II collagen antibody was a mouse monoclonal antibody (Cat. No. CP18, dilution 1:100; Millipore). The fibronectin antibody used was a mouse monoclonal antibody (MAB88916 or Roche 1087, 720; Millipore), which reacted with the human fibronectin cell attachment fragment. The antibody for integrin β1 (sc‐9970; Santa Cruz Biotechnology, Inc., Heidelberg, Germany) was a mouse monoclonal antibody against full‐length integrin β1 of human origin. Antibody combinations, characteristics, and sources are summarized in Table 1. Immunohistochemical procedures on cochlear sections have been described in previous publications (Rask‐Andersen et al., 2012). Briefly, sections were incubated on slides with a solution of the antibodies under a humid atmosphere at 4°C for 20 h. After rinsing with phosphate buffer solution (PBS) (3 × 5 min), the sections were subsequently incubated with secondary antibodies conjugated to Alexa Fluor 488 and 555 (Molecular Probes, Carlsbad, CA, USA), counterstained with the nuclear stain 4′,6‐diamidino‐2‐phenylindole dihydrochloride (DAPI) for 5 min, rinsed with PBS (3 × 5 min), and mounted with a VECTA SHIELD (Vector Laboratories, Burlingame, CA, USA) mounting medium. The sections used for antibody control were incubated with 2% bovine serum albumin (BSA), omitting the primary antibodies.

**TABLE 1:** Antibodies used in the study.

Antibody	Mono/poly	Dilution	Host	Cat. no.	Company
EMILIN‐2	Polyclonal	1:50	Rabbit	HPA040739	Sigma
Elastin	Monoclonal	1:50	Mouse	MAB2503	Millipore
Fibronectin	Monoclonal	1:50	Mouse	MAB88916	Millipore
Integrin‐β1	Monoclonal	1:50	Mouse	sc‐9970	Santa Cruz
Tenascin	Polyclonal	1:50	Rabbit	sc‐20,932	Santa Cruz
Laminin‐β2	Monoclonal	1:100	Rat	05–206	Millipore
Type IV col	Polyclonal	1:10	Goat	AB769	Millipore

### 2.2 Field emission scanning electron microscopy (FE‐SEM)

Archival SEM material was reused, as presented in earlier reports (Glueckert et al., 2005; Liu, Atturo, et al., 2015; Liu, Edin, et al., 2015; Rask‐Andersen et al., 2017). Two specimens that had undergone preparation for FE‐SEM were reanalyzed. Both were from middle‐aged females with normal hearing. Cochleae were decalcified in 0.1 M sodium‐ethyl‐diamine‐tetra‐acetic‐acid (Na‐EDTA), pH 7.4, for 6 weeks. Subsequently, they were washed in PBS, pH 7.4, dehydrated in graded ethanol (70%, 80%, 90%, 95%, and 100%; 10 min each), critical point‐dried, and attached to aluminum stubs. Specimens were coated in a BALTECH MED020 Coating System with gold–palladium to a nominal depth of 10–12 nm and viewed with a ZEISS DSM982 Gemini field emission electron microscope operating at 5 kV. Maximal resolution at this voltage was estimated to be ~ 2 nm. Digital photos were taken at 1280–1024 ppi resolution. Measurements were performed using the image analysis software Image Pro 4.5.1.29 (Media Cybernetics, Inc., Rockville, MD, USA).

### 2.3 Light and transmission electron microscopy (LM and TEM)

An archival specimen (112A, Table 2) was used to analyze the ultrastructure of the organ of Corti (OC), TCL, and BM, which also included the frequency region around 2 kHz. A semi‐thin, mid‐modiolar section in a cochlear specimen (NDL74) was cut for high‐resolution LM (Giese et al., 2024). This allowed for a direct comparison of the architecture of the OC and cochlear partition at different frequency locations. Two specimens obtained after perilymph perfusion were also analyzed using TEM. Another human cochlea obtained at surgery was analyzed using TEM (HRA75). The cochleae prepared for LM and TEM were transferred to a fixative solution containing 2% glutaraldehyde, 0.05 M sodium phosphate buffer, and 13.3% fluorocarbon to increase oxygenation and cell preservation. After fixation for 48 h, the specimens were decalcified in 0.1 M Na‐EDTA solution for 6 weeks followed by post‐fixation in 1% OsO4 in phosphate buffer. The specimens were dehydrated in graded ethanol, followed by placement in propylene oxide, and then embedded in epoxy resin. Semi‐thin sections were stained with toluidine blue, followed by thin sectioning of the areas of interest. The sections were stained in lead citrate and uranyl acetate and examined at 80 kV with a Tecnai™ G2 Spirit TEM (Thermo Fisher/FEI Company, Eindhoven, NL). Images were captured with an ORIUS™ SC200 CCD camera (Gatan Inc., Pleasanton, CA, USA) using Gatan Digital Micrograph software.

**TABLE 2:** Collected data of human cochlear specimens.

Specimen	Age (years)	Gender	PTT	Analysis
1	Middle‐aged	Female	Normal	LM, TEM (112A, SM)
2^a^	50	Male	Normal	LM (NDL75, SM)
3^b^	Middle‐aged	Female	Normal	FE‐SEM (SM)
4^b^	54	Female	Normal	FE‐SEM (SM)
5^b^	Middle‐aged	Female	Normal, 50 dB	Immunohistochemistry (SM)
6	41	Female	Normal	TEM (HRA75, SM)
7	57	Female	Normal	TEM (peril. perf.)
8	57	Male	Normal	TEM (peril. perf.)

Abbreviations: FE‐SEM, field emission scanning electron microscopy; LM, light microscopy; peril. perf., perilymph perfusion; PTT, pure tone threshold; SM, surgical meningioma; TEM, transmission electron microscopy.

^a^ Mid‐modiolar section (Giese et al., 2024).

^b^ Glueckert et al. (2005); Hayashi et al. (2015); Liu, Atturo, et al. (2015).

The thickness of the TCL was assessed in 13 mid‐modiolar sections from 10 different human inner ears of six different subjects. The cochleae were embedded in celloidin, cut in 25 μm thick sections, and stained with hematoxylin and eosin (H&E). Specimens were obtained from body donors who gave their consent for scientific use during their lifetime to the Department of Anatomy in Innsbruck. Segmentation of the TCL was performed manually by drawing a straight line on the upper surface of the BM and outlining the envelope of TCL cells in the scala tympani (ST). The manual outline helped to exclude attached debris or erythrocytes. A “Jython” (Python for ImageJ) script was created to simplify the measuring process. Automatic points were placed every eight pixels (1.97 μm) along the BM outline and an orthogonal vector was computed. The intersection with the TCL outline determined the vector's length. The arithmetic mean was calculated for all vectors along one BM to obtain the average thickness of one TCL. A mid‐modiolar section comprised apical, middle, upper basal, and lower basal TCLs. The highest average thickness in one section was defined as 100% and was set in relation to the other mean TCL thickness in the same section. This relative measurement avoided bias, such as different angles of sectioning or differences in histoprocessing, and provided data on TCL changes along the tonotopical axis.

### 2.4 Gene expression of *EMILIN2* , *ELN* , and *FBN1‐3*

To determine the gene expression level changes of the *EMILIN2*, elastin gene (*ELN*), and *FBN1‐3* (fibrillin 1–3 genes), we used RNA sequencing (RNA‐seq) analyses of human cochlear tissue without the vestibular system to track the profiles of up‐ and downregulation across fetal development (Steinacher et al., 2024). Eighteen human fetal tissue samples (gestational week [GW] 12 × 2, GW13 × 2, GW14 × 3, GW15 × 4, GW16 × 3, GW17 × 2, GW18 × 1, and GW19 × 1 as biological replicates) were used for RNA‐seq analysis. The detailed procedure of tissue preparation, RNA extraction, and next‐generation sequencing was described in a previous publication (Steinacher et al., 2024). Briefly, RNA extraction followed the manufacturing instructions of Ambion Trizol (Cat. No. 15596018; Invitrogen, Darmstadt, Germany) and the RNeasy Micro Kit (Ref: 74004, Qiagen, Hilden, Germany). RNA quantity, purity, and integrity were measured with a BioPhotometer plus (Eppendorf, Hamburg, Germany), with absorbance ratios and a Bioanalyzer 2100 from Agilent (Santa Clara, CA, USA). We then created 3′ mRNA sequencing libraries using the Quant Seq 3′ mRNA‐Seq Library Prep Kit (Lexogen, Vienna, Austria). Finally, RNA sequencing was performed on an ION Proton platform (Thermo Fisher, Karlsruhe, Germany), yielding 7–8 million reads per sample. Raw RNA‐seq data were preprocessed using the nf‐core/rnaseq/Nextflow pipeline (version 3.9). The description of the data quality control and processing was contained within the nf‐core/rnaseq pipeline. Reads were aligned to the hg38 reference genome using STAR and RSEM. Differentially expressed genes (log2 fold change > |1|, FDR < 0.5) were identified using the Bioconductor R package DESeq2 (version 4.2), and the results were plotted as DESeq2 Expression. Custom R scripts for generating plots were used for the analysis and RNA‐seq expression visualization. Relative expression levels were calculated using gestational Week 11 as a reference. We also analyzed the extracellular matrix glycoprotein fibrillin associated with the formation of elastic fibers. All codes are available from the corresponding author upon reasonable request.

### 2.5 Fetal specimens and ethical approval

Human specimens (from GW11 to GW19) were provided by the University College of London (UCL) and Newcastle branches of the Human Developmental Biology Resource (HDBR; http://hdbr.org): Joint MRC/Wellcome Trust (Grant No. MR/R006237/1). Fetal and embryonic tissues were collected with informed consent and distributed to research projects under ethical approval 18/NE/0290 from the North East‐Newcastle & North Tyneside 1 Research Ethics Committee for HDBR Newcastle and 18/LO/022 from the Fulham Research Ethics Committee for HDBR UCL London. Embryologists certified that the specimens did not exhibit visible malformations, and their embryological stages were differentiated by quantifying characteristics such as crown–rump length, external and internal morphology, and the estimated gynecological age. All specimens were devoid of any external or internal congenital defects.

### 2.6 Imaging and photography

The stained sections were examined with an inverted fluorescence microscope (Nikon TE2000; Nikon, Japan) equipped with a spot digital camera with three filters (for emission spectra maxima at 358, 461, and 555 nm). Both the microscope and camera were connected to a computer system equipped with image software (NIS Element BR‐3.2; Nikon), including image merging and a fluorescence intensity analyzer. For laser confocal microscopy, the same microscope, which was equipped with a laser emission and detection system with three different channels, was used. The optical scanning and image processing tasks were run using the Nikon EZ‐C1 (v. 3.80) program, including the reconstruction of *Z*‐stack images into projections or 3D images.

## 3 RESULTS

The human BM consists principally of four layers, as determined by electron microscopy (SEM and TEM) and immunohistochemistry: (1) epithelial basal lamina (or basement membrane) (positive for laminin‐β2 and collagen IV); (2) BM “proper” (composed of mostly radial fibers expressing collagen II and XI); (3) collagen IV layer; and (4) a cell‐rich TCL (expressing collagen IV, fibronectin and integrin‐β1) (Liu, Atturo, et al., 2015). The human BM is generally believed to contain only a thin amorphous component throughout its length, reflecting its low‐frequency responsiveness, while rodents have a relatively thick amorphous component especially at the basal end. Remarkably, high‐resolution microscopy revealed that at high frequencies (≥9 kHz), the human BM presented morphological similarities to several laboratory animals consisting of a pars arcuata and pars pectinata (Giese et al., 2024). These parallels also include the general architecture and cell anatomy of the OC.

Synchrotron radiation phase‐contrast imaging (SR‐PCI) and 3D reconstruction matching a histological semi‐thin section of a surgically obtained cochlea are shown in Figure 1a,b. Octave bands were constructed using Greenwood's formula (Greenwood, 1961). At high frequencies (>9 kHz), the BM contained a pars arcuata and a pars pectinata (Figure 2a,b). The BM is thick and consists of a radial collagen layer and a band of GS facing the ST containing few cells and occasionally dendritic macrophages. TEM revealed that the GS layer of the BM facing the ST was bordered by a thin layer of TCL cells (Figure 2c). Associated electron‐dense conglomerates in the GS were noted. GS also appeared among the bundles of collagen fibers in close association (Figure 2d).

**FIGURE 1:**
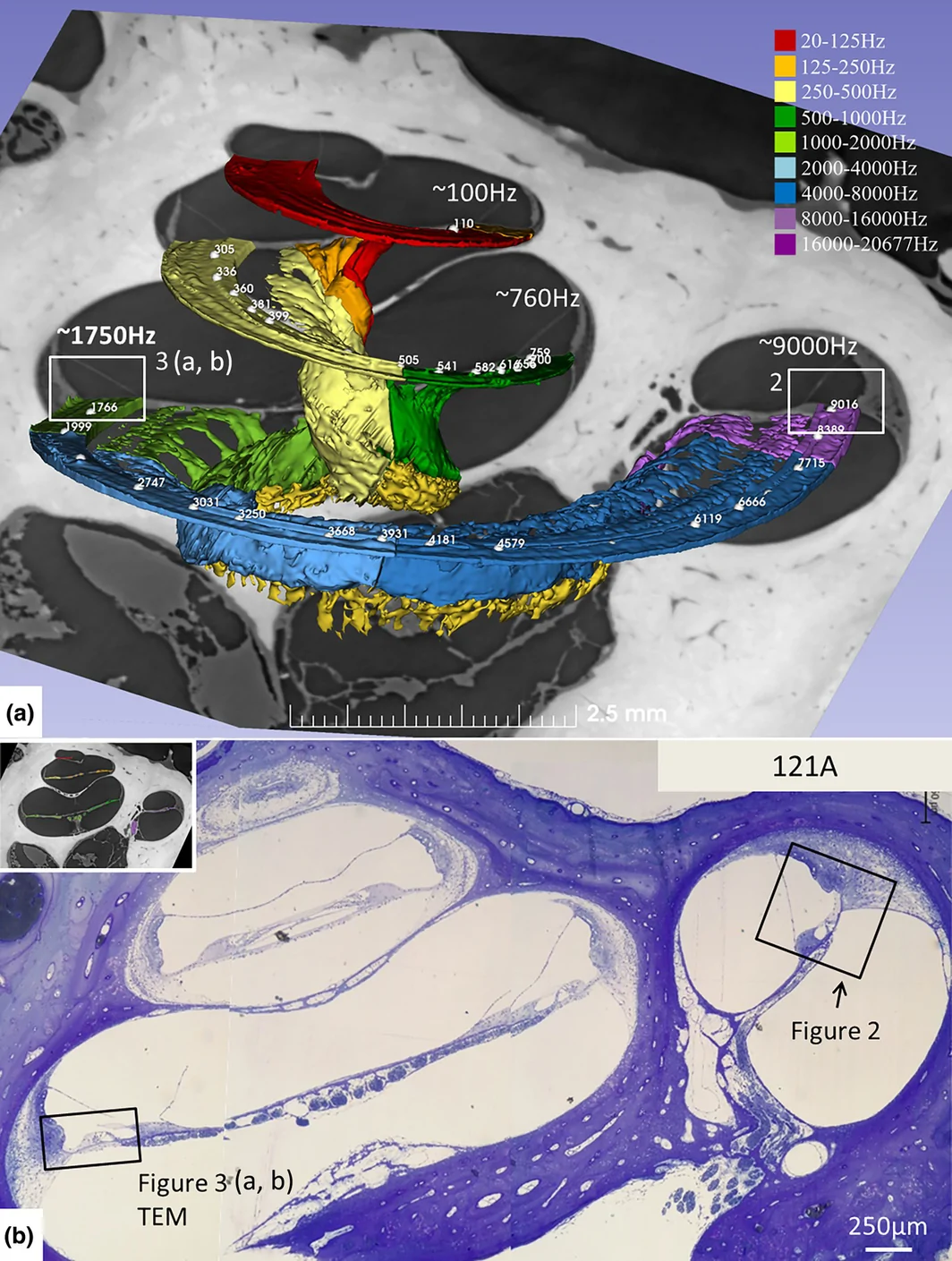
(a) SR‐PCI section and 3D reconstruction of a matched histological section of a surgically obtained human cochlea, shown in (b) (Specimen 1). Frequency bands were constructed using Greenwood's formula (Greenwood, 1961). Framed areas represent estimated frequencies at different tonotopy levels (~1750 Hz and ~9000 Hz). High magnifications at each level are shown in Figures 2 and 3. Inset in B shows the matched SR‐PCI section. Toluidine blue staining was used.

**FIGURE 2:**
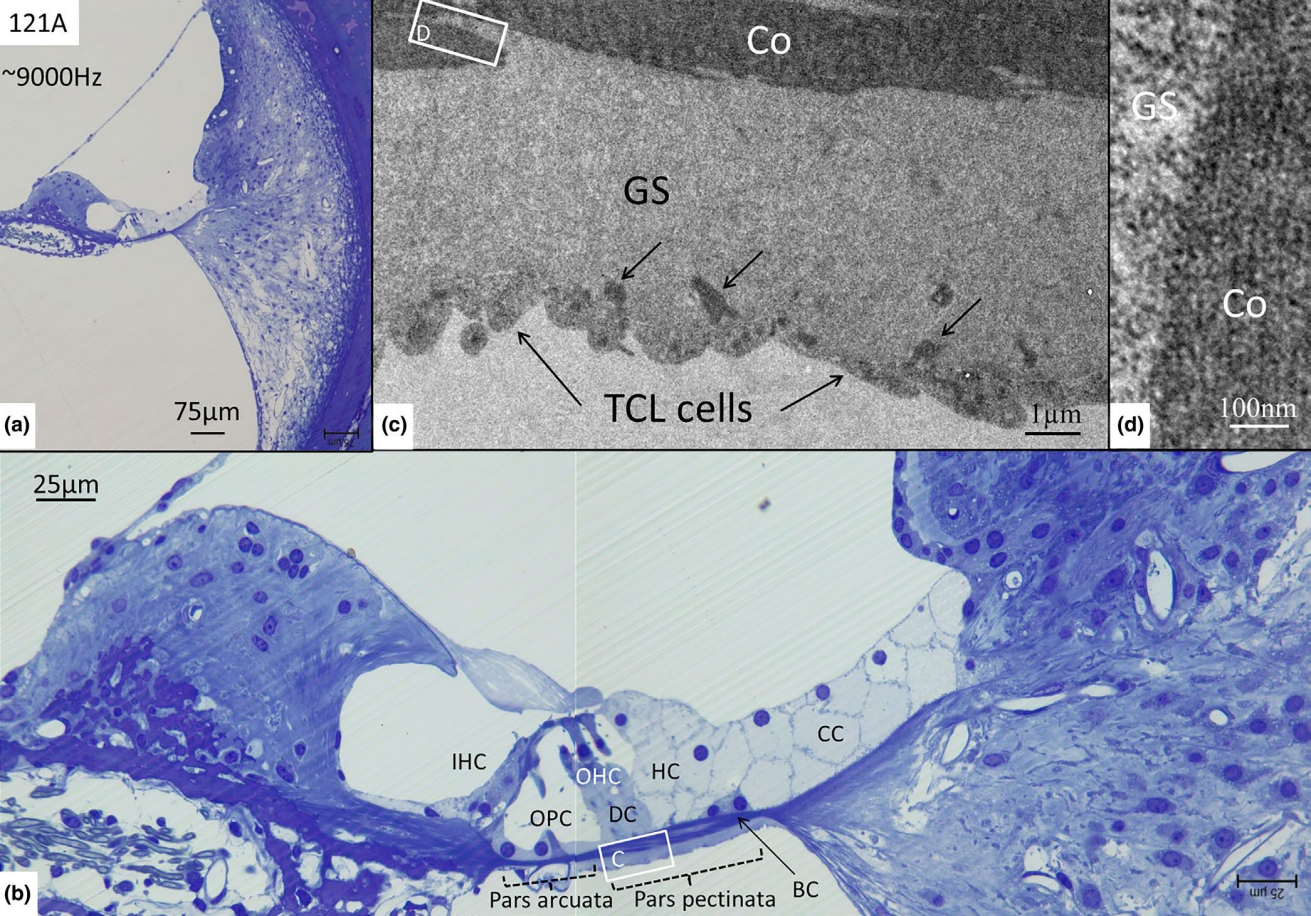
(a) LM of the cross‐sectioned basal turn in the ~9 kHz region is shown in Figure 1b. (b) Higher magnification of the OC shows the BM consisting of a pars arcuata and pectinata (staples), which contain both a layer of radial collagen fibers and a GS. (c) TEM of framed area shown in (b). The GS surface beneath the layer of collagen is margined by TCL cells. Adjacent electron‐dense areas (arrows) may represent collagen. (d) Higher magnification of the interface (framed area in (c)) between collagen (Co) and GS of the BM shown in (c). BC, Boettcher cells; CC, Claudius cells; DC, Deiters cells; HC, Hensen cells; IHC, inner hair cell; OHC, outer hair cell; OPC, outer pillar cell; TCL, tympanic covering layer (Specimen 1).

LM and TEM of the BM in the 1750 Hz region are shown in Figure 3a,b. A thick TCL faces the BM, and there is also a GS layer. Confocal immunohistochemistry of the corresponding region displays the expression of EMILIN‐2 in TCL cells and the BM proper. The inset in Figure 3c demonstrates the expression of EMILIN‐2 together with collagen II. In the middle turn, EMILIN‐2 was richly expressed in the TCL and partly associated with collagen II (Figure 3d,e). There was no EMILIN‐2 immunoreactivity in the OC.

**FIGURE 3:**
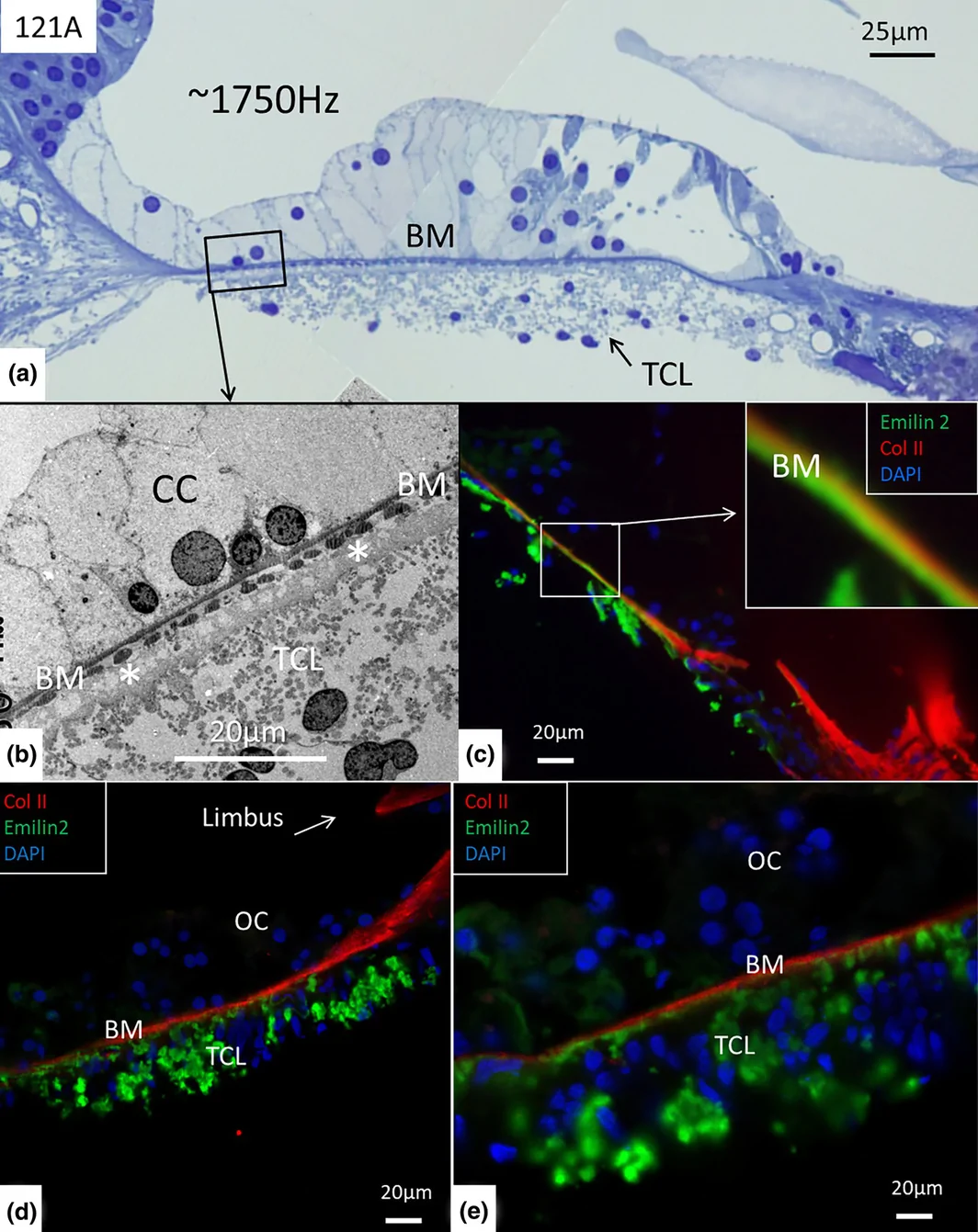
(a) Tangential section in the 1750 Hz region displayed in Figure 1. (b) TEM image of the framed area in A. The TCL cells adhere to a layer of GS (*). The area also contains electron‐dense collagenous bundles (so‐called acoustic springs). (c–e) Confocal immunohistochemistry of the TCL and the BM at a corresponding location confirmed the expression of EMILIN‐2. The framed area in (c) is magnified in the inset and demonstrates the expression of both EMILIN‐2 and collagen type II in the BM. (d, e) EMILIN‐2 is expressed in the TCL and partly in the BM proper in the low/middle frequency region (Specimen 5).

High‐resolution LM showed different morphology and thicknesses of the human BM in a mid‐modiolar section at various frequencies (Figure 4). Frequency locations were assessed by 3D synchrotron reconstruction (Giese et al., 2024). Toward the apex, the BM was wider and generally thinnest beneath the OPC and OHC regions. At 20–125 Hz, the BM thickness at the OPC foot was estimated to be 0.81 μm (Figure 4a), while at 8–13 kHz, it was 1.80 μm (Figure 4e).

**FIGURE 4:**
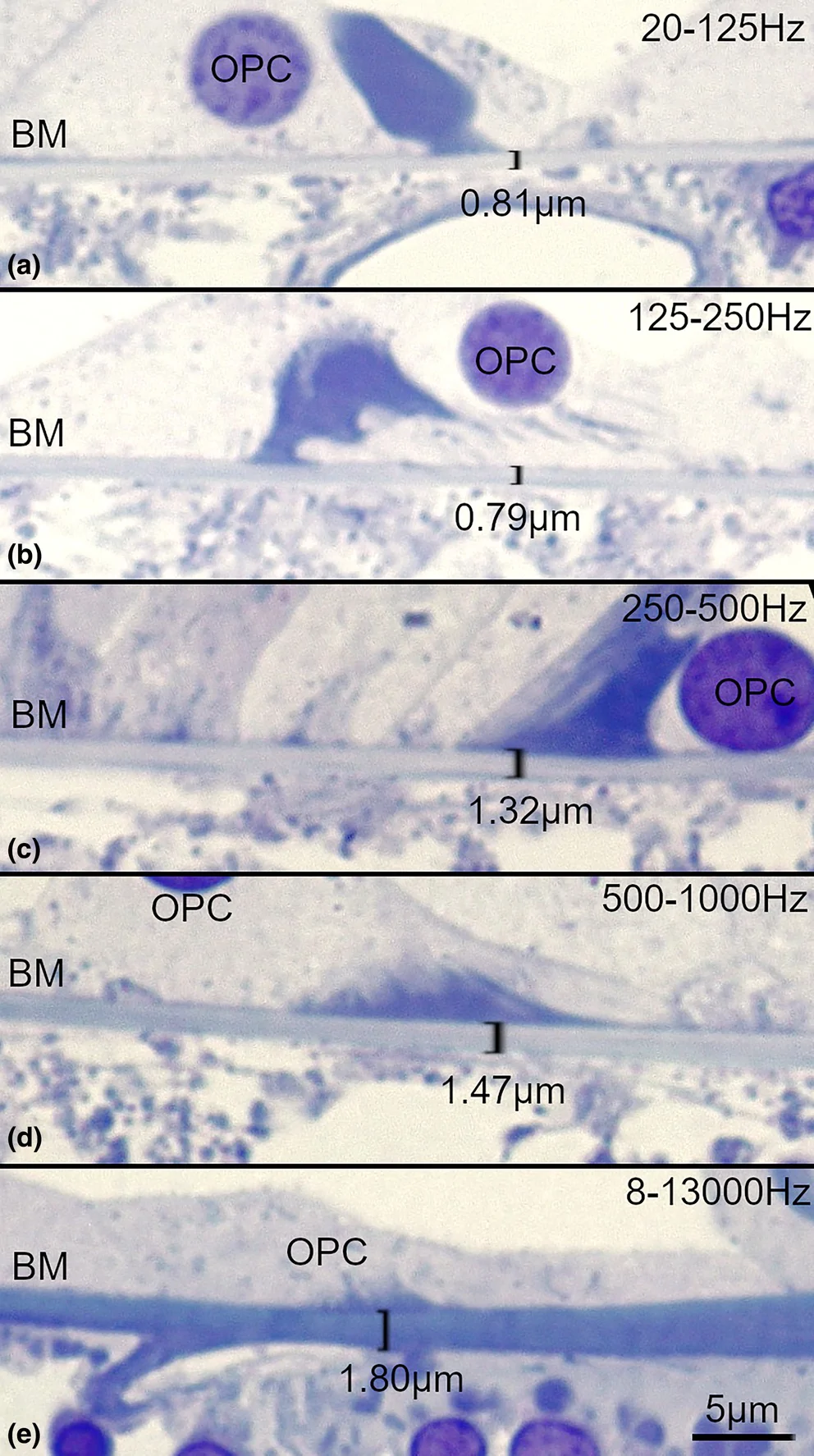
(a–e) Light microscopy of the BM at different frequency locations was assessed using a matched 3D synchrotron reconstruction of a human mid‐modiolar section (Specimen 2). The thickness of the BM at the outer pillar cell (OPC) foot below 1 kHz was less than 1 μm. At high frequencies (e), the BM is thick and consists of pars arcuata and pars pectinata. The 2–4 kHz region was not included in this specimen.

The relative thickness of the TCL at various turns assessed in 13 mid‐modiolar sections from 10 human inner ears of six different subjects embedded in celloidin is presented in Figure S1. It shows that at the apex, the TCL thickness is around 65% of the maximal thickness frequently positioned in the upper basal turn.

TEM and SEM also showed that the thickness of the TCL increased apically but diminished at the upper apical turn (Figures 5 and 6). The TCL contained many free cells with multiple long and short processes, often interconnected through adherent junctions (Figure 7). Occasionally, a thin free mesothelial cell (MC) outlined the ST side of the BM (Figure 5a,b). There was an indistinct GS that was more prominent at high frequencies. Some free dendritic cells represented macrophages. The thin basal lamina (BL) had a variable shape, sometimes fragmented or multilayered, occasionally showing an enfolding between Claudius cells (CCs) filled with a homogenous substance. FE‐SEM of a cross‐sectioned BM at 250–500 Hz showed a thick TCL layer with multiple cell processes (Figure 5c). A notable finding was the porous surface facing the ST. TCL expressed EMILIN‐2 in TCL and BM (Figure 5d). FE‐SEM of TCL at 125–250 Hz is demonstrated in Figure 5e (Specimen 4).

**FIGURE 5:**
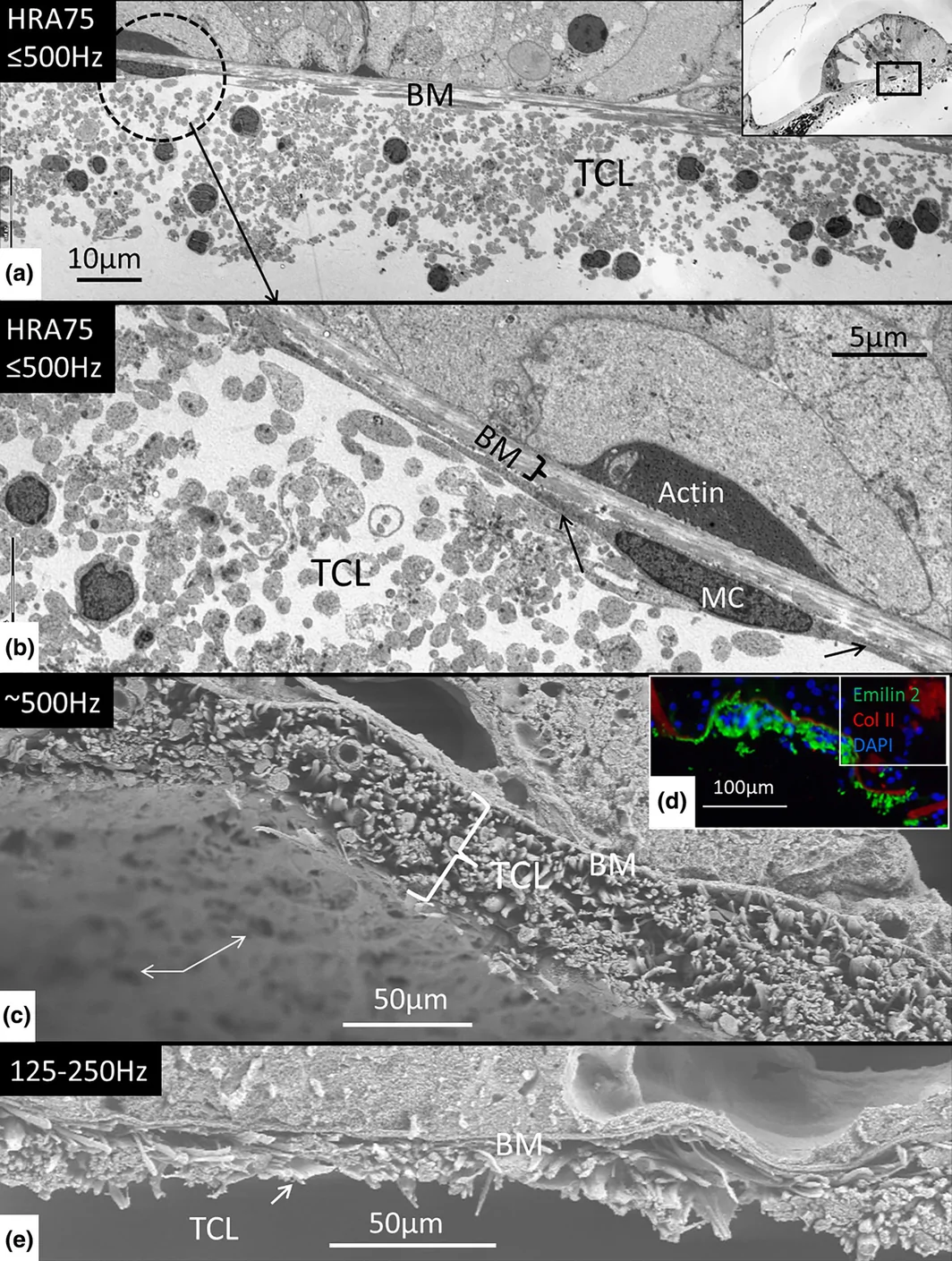
(a) TEM of the human BM and TCL in the ~500 Hz region (Specimen 6). The TCL contains free cells without any distinct intercellular GS. The encircled area is magnified in (b). The OPC foot contains actin. A thin mesothelial cell (MC) adheres to the BM. The BM consists of collagenous electron‐dense and electron‐lucent areas with a diameter of 1.25 μm. (c) FE‐SEM of the cross‐sectioned cochlear middle turn (~500 Hz region; Specimen 3). A thick TCL faces the ST, which is fenestrated (arrows). (d) TCL and BM in this region express EMILIN‐2. (e) FE‐SEM of TCL in the 125–250 Hz region.

**FIGURE 6:**
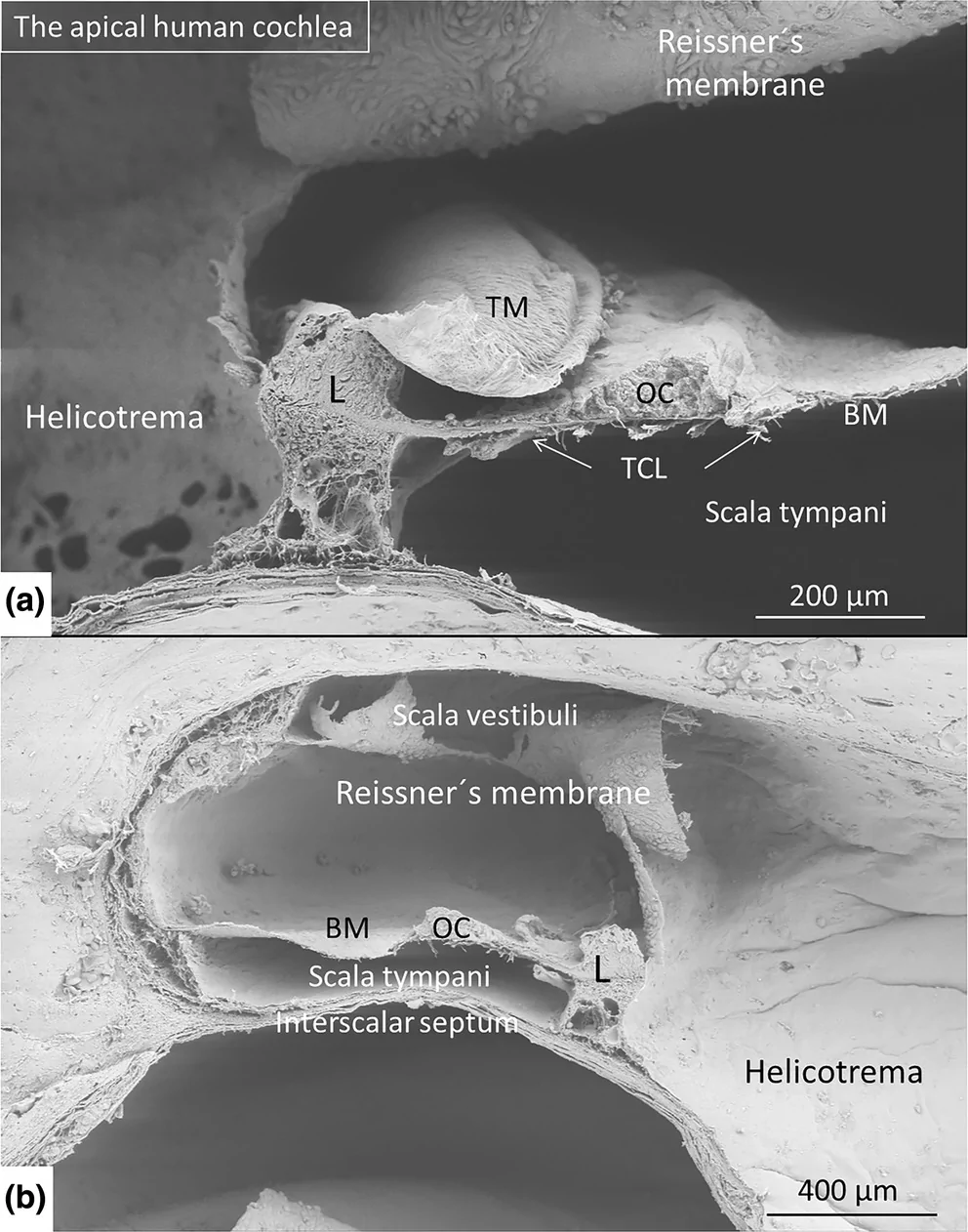
(a) FE‐SEM of cross‐sectioned apical cochlea at the helicotrema (Specimen 4). Beneath the BM, the TCL consists mostly of cross‐sectioned fibrous structures (Hayashi et al., 2015). (b) FE‐SEM of the opposite surface showing the distal cul‐de‐sac of the endolymphatic space. The BM is thin and flaccid. L, spiral limbus; OC, organ of Corti; TM, tectorial membrane.

**FIGURE 7:**
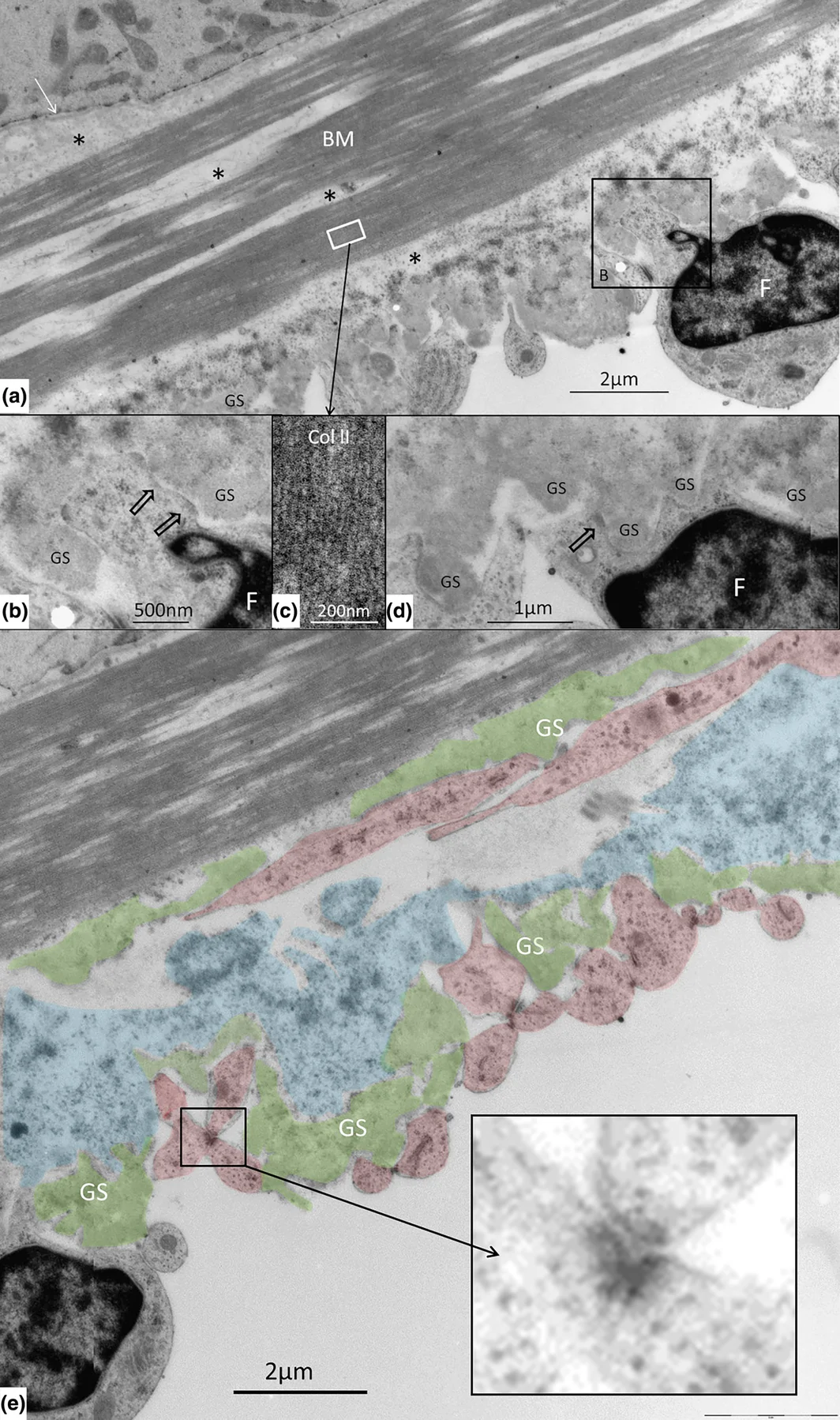
(a) TEM of BM and TCL in the high‐frequency region. The BM consists of 10–15 nm thick radially arranged fibers separated by electron‐lucent regions (*) believed to represent elastic components. Fibroblast‐like cells (F) adhere to the BM, with signs of deposition of homogenous GS (b, d). (c) White frame shows lamellar collagen fibers in higher magnification. Black frame is magnified in (d). Arrows in (b) and (d) denote focal densities (Specimen 7). (e) The TCL cells forms a layer (red) that border the BM, interconnected by adherent junctions. It is associated with a homogenous GS (green) and a fibrous matrix (blue), which are apparently secreted by the TCL cells. The framed area is magnified in the inset and shows TCL interconnected cells (Specimen 7).

In one specimen fixed with perilymph glutaraldehyde perfusion after death (Specimen 7), the TCL cells in the basal turn were interconnected by adherent junctions and formed a cell wall against the ST surface of the BM (Figure 7). These cells invariably adhered to the BM proper with deposition of homogenous GS and fibrous material into the extracellular space (Figure 7a,b,d). Lamellar BM collagen layers (Figure 7c) were separated by electron‐lucent areas. The interconnected TCL cells were closely associated with the GS as well as a fibrous matrix, which seemed to be secreted by the TCL cells showing several areas of membrane specializations (Figure 7e).

TEM and SEM exposed a multitude of fibrils with different dimensions associated with the BM (Figure 8a–c). Thin lateral fibers are shown at the basilar crest in Figure 8a. Networks with an individual fiber diameter around 40–45 nm were closely associated with thicker radial arrangements of collagen bundles having a diameter of around 80 nm (Figure 8b). Higher magnification shows a fiber meshwork with 15–40 nm fibers assumed to represent elastic microfibrils (Figure 8c). Moreover, TEM indicated that the cytoplasm of the TCL cell processes exhibited ~12 nm‐sized fibrils and microtubule structures having a diameter of around 25 nm (Figure 8d insets, Specimen 8). These fibrils surrounded the TCL cells, and the extracellular matrix also contained fibers having a diameter of around 25–50 nm that were closely associated with the TCL cell processes facing the BM (Figure 8e).

**FIGURE 8:**
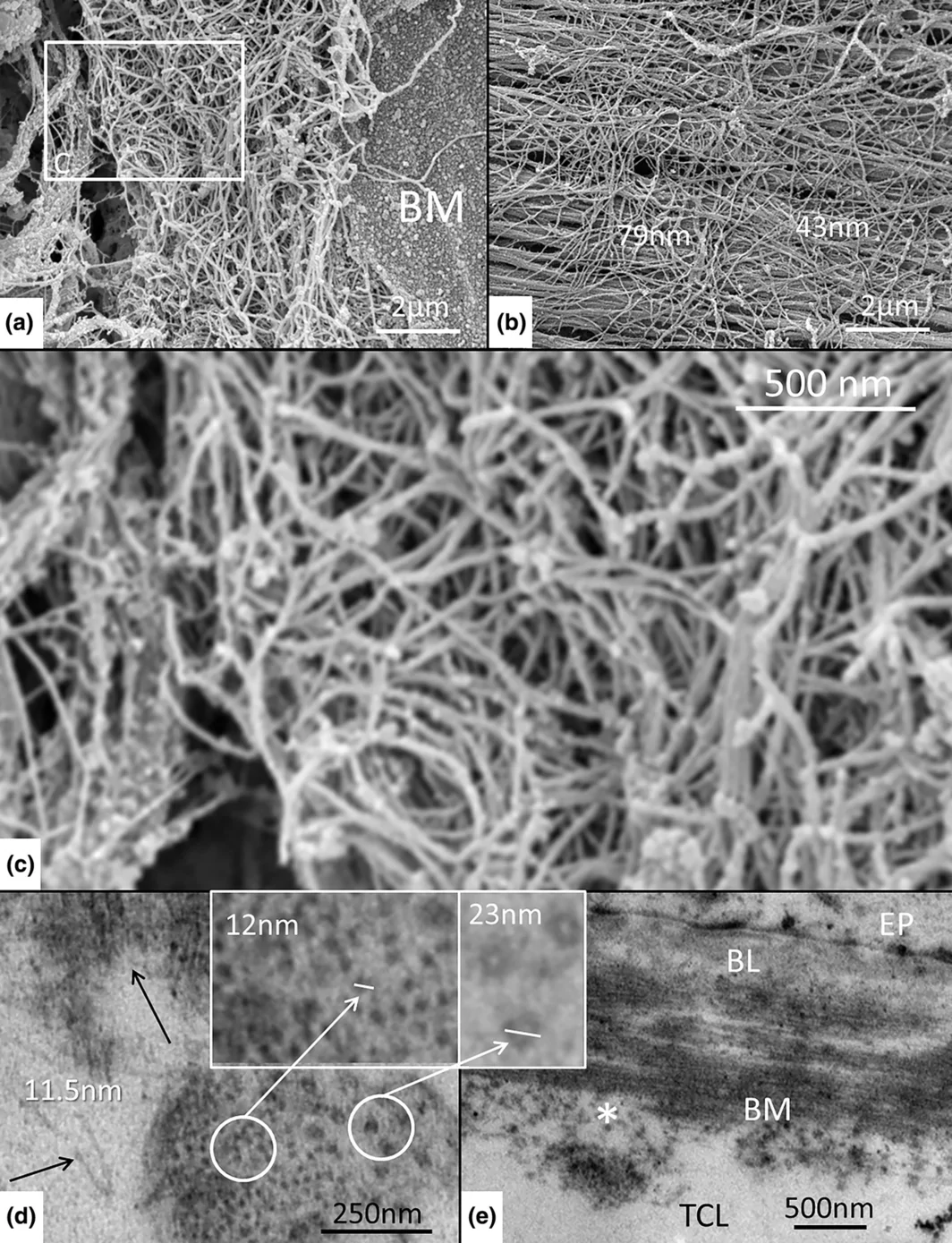
(a) SEM of human the BM at the basilar crest (Specimen 3). A higher magnification of the framed area is shown in C. (b) Meshwork of fibers with a diameter of around 40 nm intermingle with larger radial collagenous fibers (frequency~500 Hz). (c) Higher magnification of the framed area displayed in (a) shows a fiber meshwork with 15–40 nm‐sized fibers. (d) TEM of a TCL cell process beneath the BM (Specimen 8). Encircled areas are magnified in insets showing 12 and 23 nm‐sized fibrils and microtubules in the cell cytoplasm. Several 11.5 nm fibrils appear in the extracellular matrix (arrows) near the TCL cell. (e) TEM of 25–50 nm‐sized fibrils located at the inferior margin of the BM (*). BL, basal lamina; EP, epithelium.

Fibronectin was also expressed in the TCL and BM, as well as integrin 1β in the TCL, as shown previously (Liu, Atturo, et al., 2015) (Figure 9a and insets). Elastin was expressed in the BM and capillaries beneath the BM (Figure 9b,c). In the habenular region, elastin expression occasionally extended into the lateral intercellular spaces of the Claudius cells with septate projections (Figure 9d,e). A positive weak staining of tenascin was noted in the TCL (not shown here).

**FIGURE 9:**
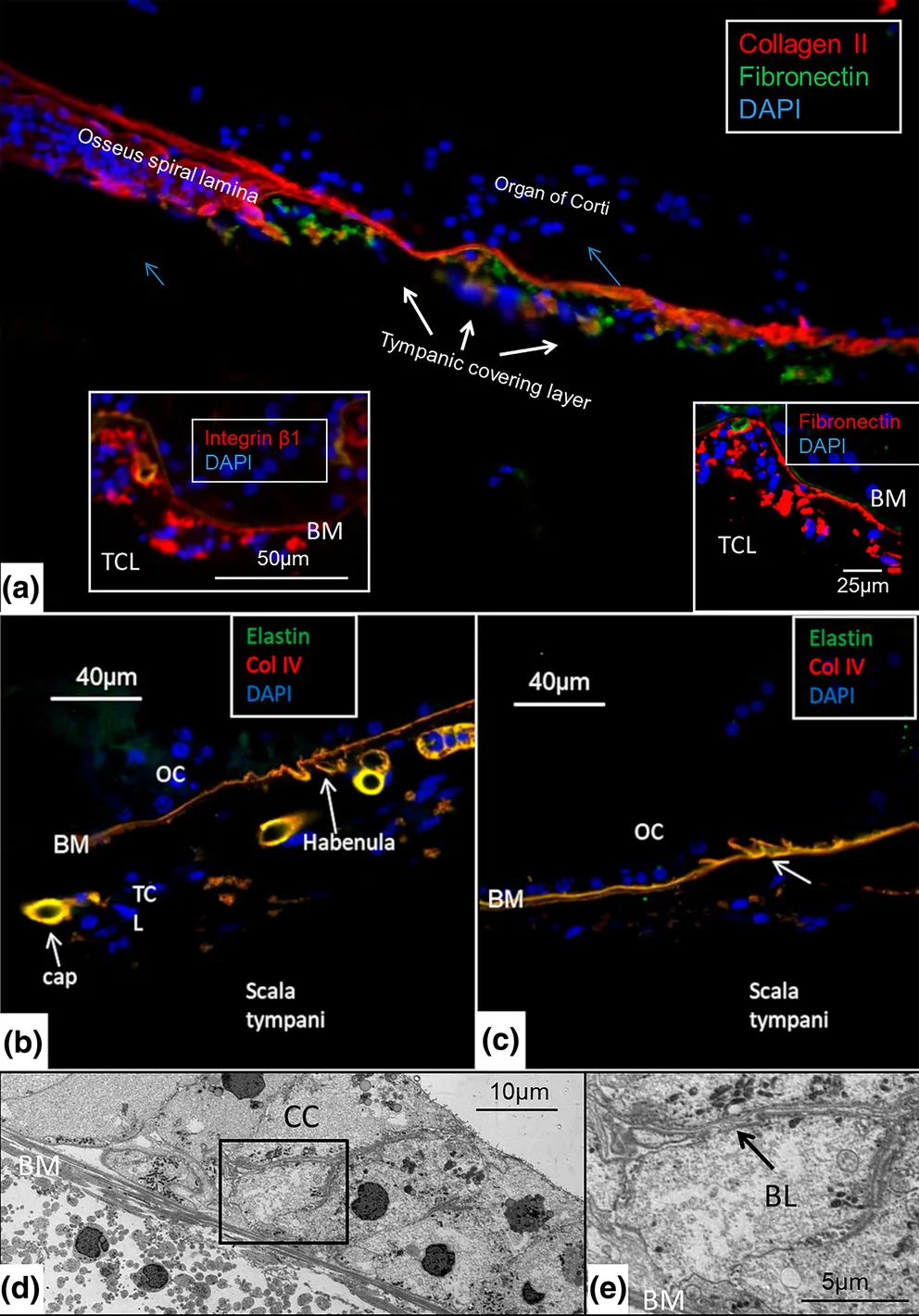
(a) Expression of fibronectin (green) and collagen type II (red). Integrin β1 is expressed in the TCL (red in left inset). Strong expression of fibronectin is shown in right inset (red). (b) BM co‐expresses collagen II and elastin in the apical human cochlea. Elastin is expressed in the BM, TCL as well as in blood vessels. (c) The BM and BL show invaginations at the habenula perforata and more laterally beneath the CCs (septa) (Specimen 5). TEM images (d) and (e) demonstrate enfoldings of the BL between the CCs (Specimen 6) (insets in (a) from Liu, Atturo, et al., 2015).

Expression of *EMILIN2*, *ELN* (encoding elastin), and *FBN1‐3* genes (encoding fibrillin‐1, −2 and −3 subtypes) from RNA‐seq data of human fetal cochlear tissue are shown in Figure 10. DESeq2 expressions are displayed as bar plots in correlation with gestational week 11 (GW11). The RNA‐seq profile is represented by differentially expressed genes (DE expression) (*y*‐axis) and gestational week (*x*‐axis). There was a downregulation of *EMILIN2* and *ELN* from GW12 to GW19. The main production in humans appears early. Fibrillin is a large extracellular matrix glycoprotein essential to elastic fiber formation. *FBN1* and *FBN2* RNA‐seq data (Figure 10b) revealed a relatively stable expression during development, although at GW 17 and 19, there was a small peak. *FBN3* gene data showed a relative upregulation of the expression profile during development with the highest peak at GW19.

**FIGURE 10:**
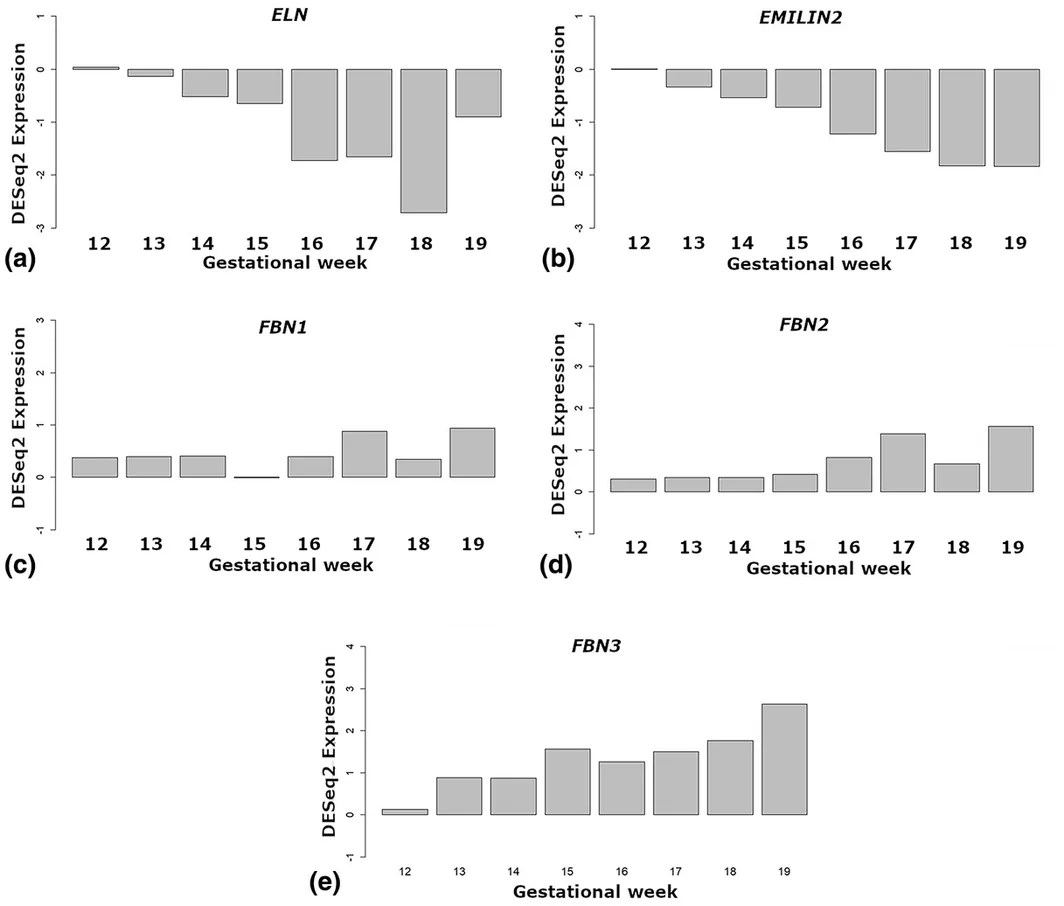
Evaluation of *EMILIN2*, *ELN*, and *FBN1–3* RNA‐seq data of human fetal cochlear tissue from GW12–19. GW11 is taken as the experimental calibrator for RNA‐seq data. The RNA‐seq profiles are represented as bar plots of differentially expressed genes (DESeq2—*y*‐axis) relative to the GW (*x*‐axis). The main production in human fetuses appears quite early. *FBN1* and *FBN2* RNA‐seq data revealed a relatively stable expression during development, with a small peak at GW 17 and 19. *FBN3* showed an increased expression profile during development with the highest peak at GW19.

## 4 DISCUSSION

More information is needed about human BM frequency‐dependent molecular composition and heterogeneity, especially for low‐frequency regions, due to their significance for human speech perception. The fundamental differences in cochlear anatomy at the base and apex seem paralleled by the differences in response coding of high‐ and low‐frequency sounds. Low‐frequency hearing may work differently from high‐frequency, not involving a place code, as observed experimentally in guinea pig models analyzed by non‐invasive optical coherence tomography (OCT) techniques (Burwood et al., 2019, 2022; Guinan, 2017; Recio‐Spinoso et al., 2017; Temchin et al., 2012; Warren et al., 2016). Processes other than cochlear amplification are thought to contribute to tuning in low‐frequency regions of the mammalian cochlea, such as timing of action potentials (Moore, 1973), velocity dependence of IHC stereocilia deflections (Dallos, 1992; Hakizimana & Fridberger, 2021), or bundle adaptation of mechanical‐electrical transducer currents (Fridberger et al., 2006).

### 4.1 EMILIN‐2 and collagen are present in the human BM

Studies in laboratory animals indicate that collagen II is present in the BM (Dreiling et al., 2002; Thalmann, 1993; Tsuprun & Santi, 1999), which is also supported by human analyses (Liu, Atturo, et al., 2015). Type II collagen is composed of three identical α1(II) chains forming fibrils less than 80 nm in diameter and is associated with water, proteoglycans, glycoproteins, and non‐collagenous proteins (Sell et al., 2010). The human BM contains an extracellular matrix in which the radial collagenous filaments are scattered in the GS (Cabezudo, 1978; Iurato, 1962; Liu, Atturo, et al., 2015; Santi et al., 1989). In the cat, the number of collagenous filaments decreases almost tenfold from the base to the apex, and the number of MCs increases, explaining the different motion patterns of the BM along its length (Cabezudo, 1978).

Iurato (1962) found no evidence that the BM contains elastin. In our previous investigation, we did not specifically focus on elastin in the BM, but we found a moderate expression in the spiral ligament in the hook region among type III fibrocytes, including some tissue margining the BM (Liu, Atturo, et al., 2015). The present study verified that EMILIN‐2 and elastin are present in the human BM in the high‐, mid‐, and low‐frequency regions. This was corroborated by RNA‐seq analyses, indicating that they formed early during development. EMILIN‐2 seems to be present and secreted by TCL cells, being incorporated into the BM. Fibrils and amorphous GS are apparently deposited from TCL cells, which may provide significant elasticity to the different regions. Based on TEM and experimental studies by Russell et al. (2020), the electron‐lucent regions of the human BM seem to represent elastic constituents. These regions appear to increase proportionally apically as the human BM thickness declines, suggesting an increased elasticity.

EMILIN‐2 belongs to a multimerin/EMILIN family of glycoproteins previously found in the elastic fibers of the heart and other tissues (Amma et al., 2003; Doliana et al., 1999). Cochlear *EMILIN2* mRNA is expressed in TCL cells and the protein of the extracellular matrix surrounding the collagenous fibers in the BM (Amma et al., 2003). Immunofluorescence of cochlear sections of wild‐type (**+/+**) and *EMILIN2* **+/−** mice showed EMILIN‐2 protein in both the TCL and extracellular matrix of the BM, suggesting that the TCL cells may secrete EMILIN‐2 into the BM (Russell et al., 2020). EMILIN‐2 seemed to be required to organize the filamentous architecture of the BM in mice for fine resolution of cochlear frequency tuning and for monitoring stiffness gradients and precision of responses in the BM for frequency resolution (Russell et al., 2020). Deleting the extracellular matrix deteriorated the organization in the BM and altered the frequency‐to‐place map. A thinning of the BM in *EMILIN2* **−/−** mice was consistent in the apical, middle, and basal turns of the cochlea.

### 4.2 Elastin and human low‐frequency hearing

The present findings may add further understanding of the sensitivity and processing of low‐frequency sound in man. Our findings concerning the sub‐micrometer thickness and elastic components in the human BM coincide with the remarkable discoveries of pillar cell geometry, reticular lamina slope, and tympanic lip angle that may add to our capability to decode low‐frequency speech signals (Giese et al., 2024; Li et al., 2025). Psychophysical tuning curves and phase gradient recordings of otoacoustic emissions support the idea that frequency resolution in humans may be sharper than in common laboratory mammals (Shera et al., 2002; Sumner et al., 2018). Moreover, humans may resolve the frequency components of competing sounds better than other commonly studied mammals. However, these findings were invalidated by others and can be explained by methodological differences (Lopez‐Poveda & Eustaquio‐Martin, 2013; Ruggero & Temchin, 2005; Shofner et al., 2005). Nonetheless, this raises questions about the evolution of speech communication and whether the human cochlea possesses unique structural features influencing elasticity to a broader activation of the apical BM for rate‐based coding, explaining our unique properties to decode speech under various conditions.

In humans, the OC and SG tonotopic maps are closely aligned for angular depths up to ~650° (Li et al., 2025). After 450°, the SG cells are spatially compacted and congregate into a central modiolar space. It is solely the basal and initial middle turns that display a well‐defined Rosenthal's canal (RC) housing the SG cells. An averaging of sound‐evoked responses across populations of neurons was proposed to explain the capacity to discriminate the pitch of low‐frequency sounds (Burwood et al., 2022). Intriguingly, unique cellular interactions and specializations between SG cell bodies have been outlined in humans that could fulfill such purposes (Rask‐Andersen et al., 1997; Tylstedt & Rask‐Andersen, 2001). Ephaptic coupling of SGN may influence timing and synchronization of action potentials and facilitate discrimination of low‐frequency sounds and 3D localization of sound. These conditions may be critical when forming strategies for electric stimulation with auditory implants. For low‐frequency stimulation, rate‐based coding may increase tonotopic mapping in the compact apical SG, even though the tonotopic map of the OC is less compressed for potential place‐based coding (Li et al., 2025).

The BM and TCL have been studied by several investigators and their studies have included research into the molecular composition of numerous species (Angelborg, 1974; Angelborg & Engström, 1974; Iurato, 1962; Retzius, 1884; Santi et al., 1989; Tsuprun & Santi, 1999, 2001). The TCL is believed to serve a protective role for the sensory organ, since it contains macrophages showing signs of phagocytosis (Angelborg, 1974; Frye et al., 2017; Liu et al., 2021). Basally, the OC may be challenged by its proximity to infection‐prone areas in the middle ear. EMILIN‐2 also belongs to a family of extracellular glycoproteins that modulate pro‐transforming growth factor‐β processing and activation of the extrinsic apoptotic pathway, as well as regulation of Hedgehog and Wnt ligand bioavailability (Schiavinato et al., 2016, 2024). In skin wound healing, EMILIN‐1 and EMILIN‐2 are deposited along fibrillin networks, which are also fibronectin‐dependent through interaction with cell surface receptors, such as integrins, collagen, and proteoglycans. Some microfibrils are believed to represent fibrillin, and this is supported by RNA‐seq data. Fibrillin is a large extracellular matrix glycoprotein that assembles into beaded microfibrils that are essential for elastic fiber formation. A potential role of *EMILIN2* in early cochlear development may involve supporting or promoting proper cellular differentiation, as well as contributing to the persistent structural integrity of the BM. In particular, EMILIN‐2 may help maintain the BM's elasticity during the pronounced morphological re‐modeling and size growth that occur throughout development. This elastic property is likely associated with the coordinated regulation of elastin, whose expression, like that of EMILIN2, decreases according to the RNA‐seq data. The expression of EMILIN2 in the BM GS during development may provide a matrix that enables the assembly or cohesion of other membrane components (e.g., fibrillin) (Amma et al., 2003; Yoshimura et al., 2014). These networks may play a crucial role in maintaining proper elasticity, integrity, and structural support for radial collagenous fiber bundles in the BM under normal conditions and during aging. Elastic fibers consist of two morphologically different components: a centrally located amorphous structure surrounded by tubular‐appearing microfibrils (110–114 Å diameter) (Cleary & Gibson, 1983; Paniagua et al., 1983). Elastin fibers are composed of 0.1–0.2 μm thick unit fibrils. These fibrils are present individually or in bundles, and they form elastin fibers, elastin meshworks, or elastin sheets in tissue (Ushiki, 2002). Fibronectin was previously described as a major component of the amorphous GS of the BM (Keithley et al., 1993; Woolf et al., 1992), as well as integrin (Santi et al., 1989) and proteoglycans, which may stabilize and hydrate BM matrices, providing stiffness and mass gradients toward the apex (Munyer & Schulte, 1994, 1995). Fibronectin has also been shown to influence wound healing (Grinnell, 1984). The fibronectin–integrin interaction can increase inflammatory signaling in macrophages. Fibronectin was demonstrated in the human cochlea with no age‐related reduction (Keithley & Tian, 1994). Nonetheless, the number of MCs was reduced at a higher age but not associated with presbycusis. In the present study, both fibronectin and integrin were expressed in the human BM (Figure 9), and the TCL cells seemed to be responsible for the production of fibronectin. Tenascin was also found to act in combination with fibronectin, regulating epithelial and MC shape and organization. Tenascin immunoreactivity was described earlier in the MCs underlying the BM and within the fibrous matrix (Ishiyama et al., 2009; Swartz & Santi, 1999).

### 4.3 Connective tissue disease and sensorineural hearing loss

Hence, besides the elastic components, a complex molecular interaction involving several extracellular matrix proteins may act to optimize frequency filtering in human BM, as well as play a role in repair and homeostasis (Figure 11). However, our findings raise issues about vulnerability and whether physiological aging, foreign bodies (cochlear implant electrodes), or various disorders may cause disorganization of the elastic fibers influencing synthesis of structural glycoproteins (Karrer, 1961; Paniagua et al., 1983; Stadler & Orfanos, 1978), thus leading to altered BM tuning and sensorineural impairment. Hearing loss is common in individuals with connective tissue and autoimmune and elastin‐driven genetic diseases that may influence the integrity of the soft inner ear tissues, including elasticity (Duque Lasio & Kozel, 2018; Skare & de Carvalho, 2025). Different mechanisms have been proposed, including increased rigidity of the BM. Further analyses of elastic fiber production and distribution, including interaction with interstitial collagen and extracellular matrix constituents, are necessary to fully understand how BM gradient composition and stiffness are related to human frequency tuning and speech perception. Nonetheless, the TCL cells seem to play a fundamental role in the production of components in the human BM providing elasticity to optimize human cochlear frequency tuning and speech perception.

**FIGURE 11:**
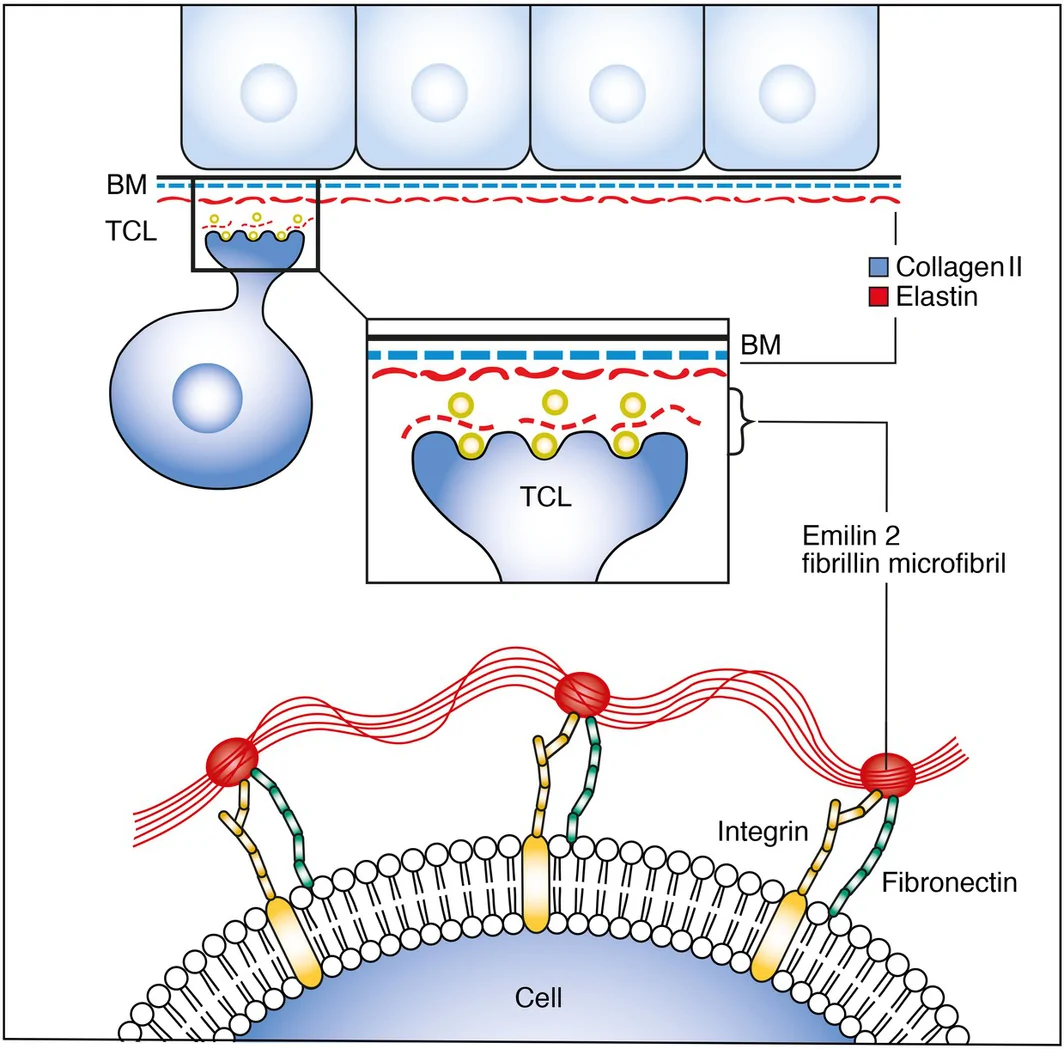
Hypothetical representation of the role of human TCL in BM formation and elasticity. EMILIN‐2 glycoprotein and matrix components may interact with fibrillin‐containing microfibrils and fibronectin/integrin to organize the assembly of elastic fibers, promoting BM sensitivity and frequency filtering. The microfibrils may form constituents of the elastic fiber, acting as a framework for the deposition of elastin (Thomson et al., 2019). (Artwork by Karin Lodin).

## AUTHOR CONTRIBUTIONS

HR‐A wrote the text of the main manuscript. WL performed the immunohistochemistry, and KS and RG conducted histological preparations and sectioning for light and electron microscopy. CS collected and analyzed immunostaining for RNA‐seq data. All authors planned the study and analyzed the results. SA, HML, and HLi processed file data and performed tonotopic mapping and analysis. All authors have read and agreed to the published version of the manuscript.

## CONFLICT OF INTEREST STATEMENT

MED‐EL Medical Electronics, R&D, GmbH, Innsbruck, Austria provided salary support for one research group member (HLi) in accordance with the contract agreement with Uppsala University, Uppsala, Sweden during 2024. The funder was not involved in the study design, collection, analysis, interpretation of data, the writing of this article, or the decision to submit it for publication.

## ETHICS STATEMENT

Human fetal and embryonic tissue (from gestational Week 12 to Week 19) was collected and distributed to research projects under ethical approval 18/NE/0290 (Approval on December 10, 2018) from the North‐East‐Newcastle and North Tyneside 1 Research Ethics Committee for HDBR Newcastle and 18/LO/022 (Approval on June 1, 2018) from the Fulham Research Ethics Committee for HDBR, UCL, London. The studies involving humans were approved by the Uppsala University Hospital [No. 99308] Ethics Review Board [Nos. 99398, 22/91999, Cont., 2003, No. C254/4; No. C45/72007, Dnr. 2013/190]. The studies were conducted in accordance with local legislation and institutional requirements. SR‐PCI samples were obtained with permission from the body bequeathal program at Western University (London, ON, Canada) in accordance with the Anatomy Act of Ontario and Western's Committee for Cadaveric Use in Research (Approval No. 06092020).

## INFORMED CONSENT

Informed consent was obtained from all subjects involved in the study. The participants provided informed consent to participate in this study. Written information was given to the patient, and informed consent was obtained (Tylstedt et al., 1997).

## Supporting information


Figure S1

## Data Availability

The original contributions presented in the study are included in the article material; further inquiries can be directed to the corresponding author. Gene expression data will be uploaded to the EBI data repository.
